# The Influence of Recycled Cement, Fly Ash, and Magnesium Oxide on the Mechanical Performance of Sustainable Cementitious Materials

**DOI:** 10.3390/ma16072760

**Published:** 2023-03-30

**Authors:** Lucas Sequeira, Blas Cantero, Miguel Bravo, Jorge de Brito, César Medina

**Affiliations:** 1 CERIS, Department of Civil Engineering, Architecture and Georresources, Instituto Superior Técnico (IST), University of Lisbon, 1049-001 Lisbon, Portugal; 2 Department of Construction Technology, University of A Coruña, E.T.S.I. Caminos, Canales, Puertos, Campus Elviña s/n, 15071 La Coruña, Spain; 3 Department of Construction, University of Extremadura, UEX-CSIC Partnering Unit, Institute for Sustainable Regional Development (INTERRA), 10003 Cáceres, Spain

**Keywords:** quaternary mortars, recycled cement, magnesium oxide, fly ash, mechanical properties, sustainability

## Abstract

In the construction industry, cement is the most widely used material. So, to achieve greater sustainability in this industry, it is imperative to improve the sustainability of this material. One way to reduce the ecological footprint of cement is to replace it, even if partially, with other more sustainable materials that can act as binders. This paper analyses the mechanical properties of more sustainable mortars containing recycled cement (RC), fly ash (FA), and magnesium oxide (MgO). Different types of binary, ternary, and quaternary mortars were used: containing recycled cement (5% and 10%), fly ash (10% and 20%), and MgO (7.5% and 15%). An experimental campaign was carried out analysing air content, density, compressive and flexural strengths, modulus of elasticity, and ultrasonic pulse velocity. The ternary mortars showed decreases between 0.4% (M-5RC10FA) and 35.3% (M-10RC15Mg) in terms of compressive strength at 365 days (compared to RM), when the theoretically expected decrease (the sum of the decreases obtained with the individual incorporation of these materials) would be between 16.6% and 41.5%, respectively. The results obtained allow for concluding that the joint use of these materials in ternary mortars improves the mechanical capacity, relative to the individual incorporation of each material in binary mortars.

## 1. Introduction

Over the past few years, there was an increase in sustainability policies around the world. The construction industry is responsible for releasing 10% of the entire amount of CO_2_ into the atmosphere, worldwide, according to the United Nations Environment Programme. To reduce the ecological footprint of Portland cement production, which represents about 5% to 7% of all CO_2_ released [1], there were several studies with materials and techniques to replace, even if partially, the amount of Portland cement needed for the manufacture of concrete. For each tonne of cement being produced, an average of 0.89 tonnes of CO_2_ is emitted [2]. Other problems arose in terms of building materials. For example, in traditional construction, some materials are scarce on islands and reefs, and using large ships to transport them long distances can cause damage to the natural environment during construction [3].

In recent times, a problem arose, consisting of excessive construction and demolition waste, and the problems it causes [4,5]. The best way to solve this situation is to try to convert this problem into a solution. Thus, some studies were carried out to use cement-based additions, starting with some concrete waste with partial incorporation of materials to replace the cement clinker. Recently, some researchers concluded that fine (<63 µm) waste from the grinding and crushing of concrete from construction and demolition waste (CDW) has chemical elements in quantities, such as those present in hydrated cement (SiO_2_ and CaO), other particles of non-hydrated cement [6,7], and calcite, which can be justified with a given ratio of the carbonated cement paste [8]. When a given fineness is reached, these compounds may contribute to cement hydration and/or have a filler effect [9]. In terms of behaviour, some studies were carried out on cement pastes with the incorporation of crushed and ground concrete as a substitute for cement. It was found that, compared to the reference pastes made with Portland cement, there was some decrease in fluidity and the initial setting time increased. It was based on these results that it was advised that the incorporation of crushed concrete waste should not exceed 15% of the total weight. Kim et al. [10] demonstrated that RC particles are larger than OPC particles, and the incorporation of RC in mortars causes lower values of compressive strength compared to reference mortars. These results are justified by a dilution effect, due to the smaller amount of cement used, compared to that used in reference mortars. Kwon et al. [11] found that the use of RC in cementitious materials allows for obtaining an overall performance of about 80% that of OPC mortars. In addition, carbon emissions can be reduced by 46%.

Kalinowska et al. [12] investigated the recycled cement, by replacing a portion of the OPC in concrete mixes. The replacement levels were 20%, 40%, 60%, and 80%. The results show that the use of recycled cement led to a reduction in compressive strength and an increase in porosity. However, the reduction in compressive strength was less pronounced for lower replacement levels (20% and 40%) and the difference in porosity was not significant between the control mix and the mixes with up to 40% replacement. In their study, the authors justified the results by pointing out that the reduction in compressive strength and increase in porosity observed in the concrete mixes with recycled cement could be attributed to the lower fineness and higher water demand of the recycled cement compared to the control cement. Overall, the study concluded that the use of recycled cement can be a viable option for reducing the environmental impact of concrete production, but caution should be applied to limit the replacement level to 40% to avoid significant reductions in compressive strength.

To increase the replacement ratio of Portland cement, the incorporation of other materials was promoted, allowing the reduction in the energy effect caused by conventional cement [13]. It is in this context that the present paper is inserted. Due to the research that was carried out to reduce the ecological footprint of Portland cement by replacing it with sustainable alternatives, cementitious materials will be produced with a low clinker ratio. Currently, the preferred clinker ratio in cementitious materials is 78%, and the main goal is to reduce it to 60% by 2050 [14].

In addition to recycled cement, fly ash was also used during the experimental campaign. The literature mentions that this material, when used in the manufacture of cementitious materials, causes a decrease in the mechanical properties, compared to the reference concretes or mortars, at the early ages of the specimens. However, with advancing age, the incorporation of FA tends to present values similar to those obtained in reference specimens without FA. This phenomenon occurs because the pozzolanic reaction is slower and longer lasting. In addition, the filler effect is quite noticeable, because the particles of FA are smaller than those of OPC, leading to a more homogeneous matrix. Chemically, the pozzolanic effect is noticeable due to the presence of siliceous compounds that, in the presence of water, are able to react chemically with calcium hydroxide [15,16,17].

Regarding MgO, Mo et al. [18] studied the effect of the use of MgO in mortars. The authors concluded that mortars with MgO incorporation present lower values of compressive strength than the reference mortar. At 90 days, the compressive strength decreased by 13.7% and 19.7% in mortars with 5% and 8% MgO, respectively. The authors attributed this reduction to less C-S-H formation due to the lower amount of cement used in mixes with MgO. Gonçalves et al. [19] incorporated relatively high amounts of MgO in the binder (10%, 15%, and 20%) in the production of cement mortars. The authors found decreases in the mechanical properties, relative to the reference mortars. In compressive strength at 28 days, the use of MgO caused a decrease between 12% and 27%. In terms of modulus of elasticity, at 28 days, there was a decrease between 3% and 12%. The author explained this trend with the higher water requirement of the mixes with MgO, which will cause the dilution of the cement due to excess water [20]. This situation results in a more porous microstructure, and therefore, a lower compressive strength. When there is a smaller amount of cement, there is a decrease in the amount of C-S-H phases that contribute to strength development [21,22], being replaced by Mg(OH)_2_, which does not have such a significant contribution to the strength [23].

Some studies were carried out on ternary and quaternary binders. It is estimated that the use of three components could decrease the pollution of the environment associated with Portland cement by 60% [24]. Following this strategy, Abdalqader and Al-Tabbaa [25] studied the compressive strength at 3, 7, 18, and 56 days of curing in cement pastes with four materials forming the binder, OPC (30–75%), FA (15–25%), ground granulated blast furnace slag (GGBS) (45–60%), and MgO (5–10%). With increasing age, the difference in compressive strength values showed a tendency to decrease. At 28 days, there was a 20% decrease compared to RP. However, at 56 days, the value of compressive strength in sustainable pastes was only 7% lower than that obtained in the reference paste. The authors justify the results with the FA properties mentioned above.

Chen et al. [26] analysed the incorporation of OPC, RC, FA, and silica fume (SF) as binders in the production of quaternary mortars. OPC was partially replaced with 30% RC, and the remaining materials were incorporated in different amounts. At 28 days, there was a greater increase in these mechanical capacities in the alternative mortars, compared to the values obtained at 7 days. The results show an increase between 30% and 90% in mechanical properties. While in the conventional mortar this increase is from 7 to 28 days was 13% in compression and 8% in flexion. This phenomenon occurs due to delayed hydration, as the pozzolanic reaction of the FA increases, originating a more compact microstructure of the mortar, leading to an increase in these properties in the long term [27,28].

Mavroulidou et al. [29] focused on evaluating the compressive strength in concrete with a mixture of three materials as binder substitutes: fly ash (FA), metakaolin (MK), and reactive magnesium oxide (MgO). A slight reduction in compressive strength was found by increasing the amount of MgO in concrete, relative to the binary mix with Portland cement and FA. This decrease can be explained by the formation of larger pores volume and, consequently, the increase in porosity of these mixes, when compared to reference concrete [20].

Unluer and Al-Tabbaa [30] investigated the compressive strength of concrete under accelerated carbonation conditions (10% of CO_2_) with MgO and FA. The results show that the strength in mixes with MgO and FA was more than twice that in mixes without FA. The strength of the mixes gradually increased with the CO_2_ concentration (from 0% to 20%).

Cantero et al. [31] studied the influence of fly ash on the mechanical properties of cementitious materials produced with recycled cement. After analysing the results, it was concluded that the mortars produced with partial replacement of OPC with recycled cement (5%) and fly ash (10%) showed similar values of compressive and flexural strength, relative to those obtained in the reference mortars, with 100% OPC. The authors also analysed the energy consumed per strength at 365 days of age. The use of RC (10%) and FA (20%) reduced the energy consumed per strength by about 13%, relative to the conventional mortar.

Despite the increase in studies on this subject lately, it is necessary to deepen the knowledge about cementitious materials with replacement, even if partial, of Portland cement with various materials with less ecological footprint. Following this reasoning, in this paper, the possibility of producing mortars with four different binders was studied: ordinary Portland cement, recycled cement (5–10% by weight), fly ash (10–25% by weight), and reactive magnesium oxide (7.5–15% by weight). In order to study the synergistic effects, different types of mortars, ternary and quaternary, were produced. The ternary mortars produced were M-5RC7.5Mg, M-10RC15Mg, M-5RC10FA, M-10RC20FA, M-7.5Mg10FA, and M-15Mg20FA. The quaternary mortars produced were M-22.5mix (5% RC, 7.5% Mg and 10%FA) and M-45mix (10% RC, 15% Mg and 20% FA).

In order to fill some gaps on this subject in the scientific community, an experimental campaign was carried out. This consisted in characterizing these sustainable mortars in the fresh state through workability, air content, and density, and in the hardened state through the following tests: compressive strength, flexural strength, ultrasonic pulse velocity, and dynamic modulus of elasticity. Finally, an analysis of the energy consumed of the mixes produced was performed. The results can open a new window for the combined use of some of these four materials as binders, namely OPC, RC, FA, and MgO.

Nowadays, there are still few studies about ternary and quaternary cementitious materials with some or all of these materials (RC, FA, and MgO). The partial replacement of OPC with more than one material is becoming increasingly important in order to reduce the amount of OPC used as much as possible and mainly to analyse the synergistic effects of these different alternatives. It is necessary to deepen this knowledge and expand the literature on this subject. Another innovative point in this research is the testing of mortars after 365 days of curing. Long-term tests are not usual, but it is very important to understand the behaviour of these new mortars at this age.

## 2. Materials and Methods

### 2.1. Materials

Several materials were used to produce the mortars. Fine and coarse silica sands were used. These fine and coarse sands have, respectively, a dry density of 2637 kg/m^3^ and 2617 kg/m^3^, according to the EN1097-6 standard [32]. Fine and coarse sands have water absorption of 0.4% and 0.5%, respectively.

A reference mortar (RM) was also produced to compare the results obtained with Portland cement type I 42.5 R.

The remaining mixes incorporated fly ash, recycled concrete power, and reactive MgO. About the fly ash, a class F fly ash from EDP-Gestão da Produção de Energia, S.A. (Sines Power Plant, in Portugal) was used. The RC was obtained by grinding and crushing concrete specimens that were produced in the laboratory. The MgO was provided by the company Wisium (Spain). This magnesium oxide is considered a light-burned MgO, due to its calcination temperature (between 800 °C and 1000 °C) and has a reactivity of 3544 s.

The aggregates and binders were chemically analysed by XRF (X-ray fluorescence) using the ZSX PRIMUS IV (Rigaku) equipment with a 4 kW power.

To determine the composition of each mortar, Faury’s curve [33] was used, with 612 kg/m^3^ of binder, an effective water/binder ratio of 0.55, with an environmental exposure class of XC3, targeting an S3 workability class, according to the EN206-1 standard [34]. The mortar specimens were produced and placed in prismatic moulds with 160 × 40 × 40 mm, and 24 h after production, the specimens were demoulded. Curing took place in a saturated chamber (RH > 95%) in the laboratory for 28 days.

Table 1 presents the chemical composition of the materials used in the mixes. This table shows the amount of each oxide present in the different materials used. It is important to note that these percentages are by mass. Silica is the oxide present in larger amounts in these materials. This is natural given the siliceous origin of the aggregates. Regarding the binders, it is possible to note that in OPC, RC, and FA, the oxides present in greater amounts are SiO_2_, CaO, and Al_2_O_3_. In OPC, CaO appears at 67.1%. In RC and FA, SiO_2_ is the oxide in the greatest quantity at 47.5% and 57.5%, respectively. In MgO, as expected, the oxide with the greatest amount is MgO (84.9%). This means that the reactive MgO used in this experimental campaign has a purity of 84.9%.

The physical properties of the binders used in the production of the mortars can be observed in Table 2. The binders were analysed in terms of size, specific surface (by BET method), and density. The respective particle size distribution was obtained in a Mastersizer S. Analyser (Malvern Instruments) using ethanol as a dispersant.

Table 2 shows that, compared to the other materials, the particles of FA have a smaller size. In fact, it can be seen that 50% of the FA particles are smaller than 7.6 µm, and 90% are smaller than 43.6 µm. As mentioned, this smaller size of the FA particles may have positive consequences on the behaviour of cementitious materials with this material. This is due to the filler effect, which can lead to a more homogeneous mortar matrix, and to a pozzolanic effect. On the other hand, the larger particle size of RC and MgO can cause a negative effect of cementitious materials produced with these two materials.

### 2.2. Mix Design

As previously mentioned, the first goal of the experimental campaign carried out is to analyse the individual incorporation of RC (5–10% by weight), FA (10–20% by weight), and MgO (7.5–15% by weight), with the remaining amount of binder filled with OPC. Additionally, the main goal is to analyse the simultaneous incorporation of two or three of these materials with OPC in the production of the mortars, also in the amounts (%) indicated above. The amount of binder, as well as the water/binder ratio, remained constant in all mortar compositions at 612 kg/m^3^ and 0.50, respectively. Table 3 shows the composition of the RM. The composition of the other mixes only varies in the amount of each binder, according to the defined and explained percentages.

### 2.3. Mortar Properties

In this research, the properties of mortars in their fresh and hardened states were studied. In the fresh state, tests such as consistency, air content, and density were carried out, following standards EN 1015-3 [35], EN 1015-6 [36], and EN 12350-7 [37], respectively. Particular care was required during moulding in order to minimize pouring and setting times. After this process, the specimens were wrapped in plastic and stored for 24 h. At the end of this period, the mortars were placed in the humidity chamber (20 ± 2 °C and 95 ± 5% of humidity) until tested. In order to investigate the mechanical behaviour of these mortars, the compressive and flexural strength and dynamic modulus of elasticity were analysed. In terms of compressive and flexural strengths, the specimens were performed according to EN 1015-11 [38] on 160 × 40 × 40 mm prismatic samples. All mixes produced were tested at 7, 91, and 356 days. Dynamic modulus of elasticity tests were also performed at 91 and 365 days, following the indications of ASTM E1876-15 [39]. In addition to these tests, another non-destructive test was performed, the ultrasonic propagation velocity test, also at 91 and 365 days, according EN 12504-4 [40]. For each mix, three specimens were produced to study the mentioned properties.

Finally, the eco-efficiency of the material was analysed following the method presented by Hamidi et al. [41]. This method consists of the energy consumed in the manufacture of the binders (with 2, 3, and 4 components), considering the respective manufacturing process and the final grinding of the binder. The energy required is a function of the composition of the binders, and is given by the energy required for their manufacture and grinding (Equation (1) [41]).

(1) $$\;E\;\left(kW\cdot h/t\right)=\overset{n}{\underset{i=0}{\sum }}C_{i}\cdot \left(E_{ci}^{process}+E_{i}^{grinding}\right)$$

where *E* is the energy consumed in the production of one ton of binder and C the proportion of each material that constitutes the binder, in this case OPC (1, 0.95, 0.925, 0.9, 0.85, 0.8, 0.775, and 0.55), RC (0, 0.05 and 0.10), FA (0.10 and 0.20), and MgO (0.075 and 0.15). $E_{ci}^{\begin{array}{c} process \end{array}}$and $\;E_{ci}^{grinding\;}$ are the energies (kW·h/t) associated with the production and grinding required to achieve each type of binder, respectively. After obtaining the results from Equation (1), the energy performance for each type of mortar can finally be calculated according to Equation (2) [41], where f_cm_ is the compressive strength of the mortar.

(2) $$E{\;}_{performance}\left(kW\cdot h/t\cdot MPa\right)=\frac{E}{f_{cm}}$$

## 3. Discussion of the Results

### 3.1. Fresh-State Properties

#### 3.1.1. Consistency

Table 4 shows the consistency of the mortars produced. Analysing each material individually, different trends come up. The incorporation of RC at 5% and 10% causes a reduction in workability by 1.1% and 4.4%, respectively, relative to RM. This slight decrease is justified by the higher water absorption of this binder, and due to the presence of a larger amount of particles smaller than 10 μm, is capable of absorbing more water [42,43,44]. The use of 10% and 20% of FA, by mass, causes a 7.7% and 17.7% increase in workability, relative to the reference mortar. Analysing the mortars with the incorporation of 7.5% and 15% of MgO by weight, there is a slight decrease in consistency of 4.8% and 8.9%, compared to the RM. This slight variation is due to the fact that, despite the similarity in particle size of OPC and MgO, MgO has a high early reactivity, requiring a greater amount of water [45]. However, because the water/binder ratio remained constant, a decrease in workability is normal when MgO is used in mortars.

When RC and FA are added simultaneously (mixes M-5RC10FA and M-10RC20FA), the workability of the mortars increases by 4.8% to 11.1%, respectively, relative to the conventional mix. Furthermore, when compared to binary mortars containing only RC (M-5RC and M-10RC), an increase in workability of 6.0% to 15.8%, respectively, is observed. These results suggest that using both RC and FA simultaneously can effectively address a decrease in the workability and reduce the higher water demand associated to the use of RC [26].

In mortars M-5RC7.5Mg and M-10RC15Mg, there was a decrease in workability of 2% and 14%, respectively, compared to RM. No clear improvement in workability was observed when these materials were used simultaneously, compared to the individual incorporation. This is mainly due to the larger size of the RC and MgO particles compared to the OPC particles.

However, the M-7.5Mg10FA mortar showed better results than RM, with a 3.3% increase in workability. As with the ternary mortars with FA and RC, using both MgO and FA can effectively address a decrease in workability and reduce the higher water demand associated with using only MgO.

Finally, the mortars with the incorporation of the four materials (OPC, RC, FA, and MgO) showed a small decrease in consistency values: M-22.5mix and M-45mix had a slight decrease in workability of 2.2% and 2.6%, respectively, relative to RM. This variation is similar to that expected if one adds up the variations that occurred when incorporating each material individually, with an error of approximately only 1.4%.

#### 3.1.2. Air Content and Density

Table 4 also presents the results obtained in terms of air content and density. Firstly, analysing the binary mortars with OPC and RC, it can be seen that, when incorporating 5% and 10% of RC, there is an increase in 19% and 38% of air content, respectively, compared to RM. This is largely due to the higher porosity of the mortar mentioned above and the more irregular shape of the RC particles when compared to the OPC particles. The use of 10% and 20%, by weight, of FA leads to a decreasing trend in the order of 37.5% and 50%, respectively, when compared to RM. This result is justified by the more spherical shape and smoother surface of the FA particles compared to those of OPC. In terms of MgO, this was where there was a greater increase in air content, such that the incorporation of 7.5% and 15% of this material caused an increase of 31.3% and 75% of the mentioned test, compared to RM.

Analysing the ternary mixes, simultaneously incorporating RC and FA (mixes M-5RC10FA and M-10RC20FA) led to a reduction in air content of 31% and 57%, respectively, compared to the conventional mortar. This reduction in air content may be attributed to the filling effect of the FA particles.

On the other hand, using RC and MgO simultaneously (M-5RC7.5Mg and M-10RC15Mg) led to an increase in air content of 38% and 79%, respectively, compared to RM. These results are justified by the larger particle size of these materials, relative to OPC particles, not filling the available pores, and increasing the air content.

In mortars M-7.5Mg10FA and M-15Mg20FA, there is a decrease in this property of 25.0% and 69.0%, respectively. As previously justified, these results are justified by the filler effect of FA particles on mixes with MgO particles.

Finally, analysing the quaternary mortars, there was a decrease of 25.0% and 37.5% when they were used at 22.5% and 45% (RC, FA, and MgO), compared to the reference mortar. These results, relative to the previous ones, seem to be largely due to the use of FA in the mortar composition and the filler effect of the FA particles in the use of RC and Mg.

**Figure 1:**
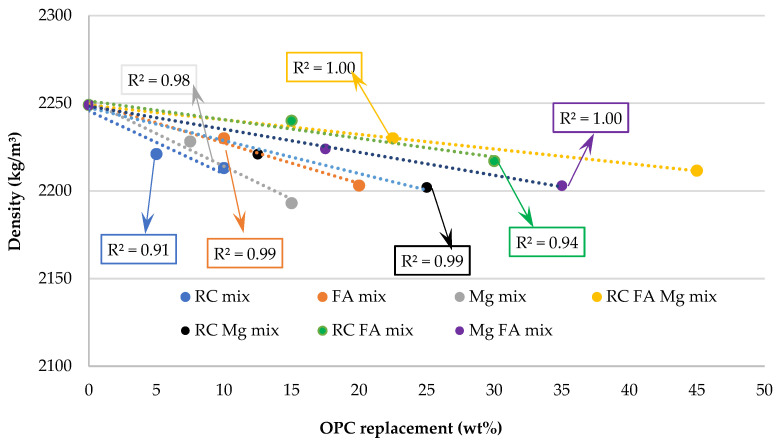
Density versus OPC replacement in the mixes.

On the other hand, the individual incorporation of RC, FA, and MgO causes a slight reduction in density (less than 2% in all mixes analysed), keeping density values within the expected range (~2200–2250 kg/m^3^) for mortars with the use of the three alternative materials used. A strong linear relationship is observed for each of the materials, as evidenced in Figure 1, by its high R^2^, which is always greater than 0.91.

Finally, in the ternary and quaternary mortars, the incorporation of RC, FA, and MgO led to density values higher than those found for the individual incorporation of each of the materials. This trend can be justified by the filler effect of the FA particles on mortars with RC and MgO particles, giving rise to cementitious matrices of higher density.

### 3.2. Hardened-State Properties

#### 3.2.1. Compressive Strength

Table 5 presents the results of compressive strength at 91 days of age for specimens of the different mixes produced. They show that the mortars with 5% and 10% of RC replacing OPC showed a decrease in compressive strength of 11.5% and 18.4%, respectively, relative to the reference mortar. These values are justified by the dilution effect and the low reactivity of the mortars with RC [10]. Regarding the incorporation of 10% and 20% of FA in the mortars, a decrease in this property is also observed, at 9.2% and 1.7%, respectively. This decrease occurs because the clinker dilution effect prevailed over the pozzolanicity of FA [46]. When incorporating 7.5% and 15% of MgO in place of OPC, the compressive strength at 91 days decreased 4.2% and 19.0%, respectively, compared to RM. This trend is because the incorporation of MgO leads to a decrease in the amount of cement, and since the hydration products of cement are more resistant than those of the oxide (Mg(OH)_2_), the greater the amount of MgO present in the concrete, the greater the reduction in compressive strength [19,20,21,22].

In terms of compressive strength, in the mortars that involve both RC and FA decreases of 13.8% (in mix M-5RC10FA) and 31.6% (in mix M-10RC20FA) were observed, compared to the conventional mortar. This fact suggests that the simultaneous use of RC and FA in mortars can improve their compressive strength compared to binary mortars that contain only RC or FA. This improvement in compressive strength is due to RC, which complements the reactivity of the SCMs (in this case, FA) [47]. Previous studies on mortars that incorporated limestone powder (10–20%) with FA (10–30%) or recycled concrete powder (10–40%) with FA and SF (15–30%) observed similar results [26,28].

When using RC and MgO together, it is not possible to draw a clear conclusion about the synergy between these two materials. For mortar M-5RC7.5Mg, a theoretical expected value lower than the one obtained experimentally was found. However, for M-10RC15Mg, a positive synergy between these two materials was observed, with the negative variation in the value obtained experimentally (relatively to the reference mortar) being lower than the one expected theoretically.

The simultaneous use of MgO and FA causes a decrease in compressive strength by 19.6% (M-7.5Mg10FA) and 43% (M-15Mg20FA) compared to RM at 91 days. These values are higher than those that would be theoretically expected. However, at 365 days, it is clear that both mixes with MgO and FA present experimentally obtained compressive strength values much lower than those that would theoretically be expected. This phenomenon occurs after 365 days, because MgO is an accelerating admixture that can speed up the hydration process of the cementitious compounds in the concrete mix. However, when used in combination with fly ash, the reaction between MgO and FA can delay the strength gain of concrete. This delay in strength gain can result in lower compressive strength at early ages [23]. Finally, analysing the results of the quaternary mortars, it was found that they suffered a reduction in compressive strength at 91 days of 19.7% and 56.6% for M-22.5mix and M-45mix, respectively, compared to the conventional mortar. Adding the reductions for each type of binary mortar, it would be expected that for mixes M-22.5mix and M-45mix, the reduction in this property would be 24.9% and 61.3%, respectively. It can be concluded that, when using the four materials at the same time, the values actually obtained are better than those obtained by the individual sum of each of them.

This simultaneous use of RC, FA, and MgO in mortars can result in the formation of multiple gels that contribute to the strength and durability of the material. FA contains silica and alumina, which can react with Ca(OH)_2_ produced by the hydration of the cement to form calcium silicate hydrate (C-S-H) gel. This pozzolanic reaction is a key mechanism that contributes to the strength of the mortar. MgO can also play an important role in the performance of the mortar. When MgO is mixed with water, it reacts to form Mg(OH)_2_, which can then react with silica or alumina in the fly ash to form magnesium silicate hydrate (M-S-H) or magnesium aluminate hydrate (M-A-H) gel, respectively. These gels are similar to C-S-H gel and can contribute to the strength and durability of the mortar. However, the carbonation of MgO can reduce its alkalinity and affect the pozzolanic reaction between fly ash and Ca(OH)_2_. On the other hand, RC may contain unhydrated cement particles that can react with Mg(OH)_2_ to form C-S-H gel, similar to the pozzolanic reaction between FA and Ca(OH)_2_. Overall, the combination of RC, FA, and MgO can lead to the formation of multiple gels, including C-S-H, M-S-H, and M-A-H, which can contribute to the strength and durability of these mortars [20,21,23,48,49].

**Figure 2:**
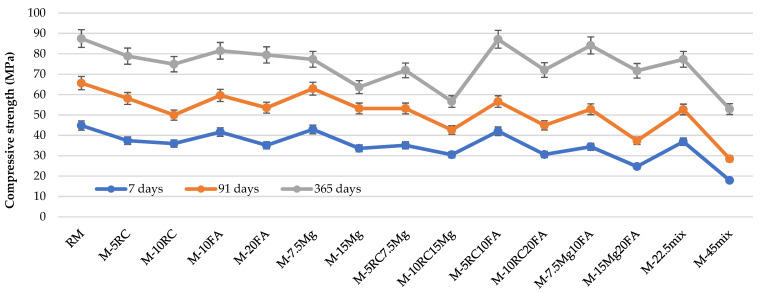
Compressive strength at 7, 91, and 365 days.

Figure 2 shows the results of compressive strength between 7 and 365 days of age for specimens of the different mixes produced. After 365 days of curing, the quaternary mortars show reductions of 11.6% (M-22.5mix) and 39.5% (M-45mix), compared to the reference mortar. It should be noted that there was a lower percentage decrease in compressive strength from 91 to 365 days of these mortars, compared to RM. This result is justified by the expansive capacity of MgO and with the refinement of the pore structure, caused by the more sustainable materials used [28].

It is also noteworthy that, at 365 days, only three of the mixes show higher variations than those denoted at 91 days. These three mixes have in common the use of MgO (M-7.5Mg, M-15Mg, and M10RC15Mg). This result is clearly related to the modifications induced by the simultaneous use of the additions, specifically in the refinement of the pore structure [21]. It is also important to observe in Table 5 that, in all ternary and quaternary mixes, the experimentally obtained variation values were lower than those theoretically expected. This shows the good behaviour of the mortars produced with age.

#### 3.2.2. Strength Activity Index

Table 6 allows for analysing the strength activity indexes (SAI) of the mortars at 7, 91, and 365 days of age of the specimens.

As previously analysed, the partial replacement of OPC with RC, FA, and MgO, either individually or in ternary/quaternary mortars, causes a decrease in the compressive strength capacity at 7 days of age, compared to RM. However, at this age, all mortars produced, except four of them, obtained SAI values higher than 75.0% (value required by ASTM C618-19 [50] for binders containing a maximum of 20% of FA).

The SAI after 7 days of curing presented values between 80% and 83% for mortars with OPC and RC, between 78% and 93% for mortars with FA and OPC, and values between 75% and 96% for the use of MgO and OPC. For quaternary mortars, values of 82.3% and 39.9% were obtained for M-22.5mix and M-45mix, respectively. M-45mix is one of the three mortars that did not reach the value established for the mentioned standard at all ages analysed. The results obtained are in agreement with those obtained by Horsakulthai [51], who found SAI values at 7 days higher than 89%, in mortars containing a maximum of 20% of RC.

After 365 days of curing, it was found for M-22.5mix a SAI of 88.4%, a value seen as quite positive. It was also possible to observe an increase in the SAI value with the age of the test in almost all mortars produced.

#### 3.2.3. Flexural Strength

**Figure 3:**
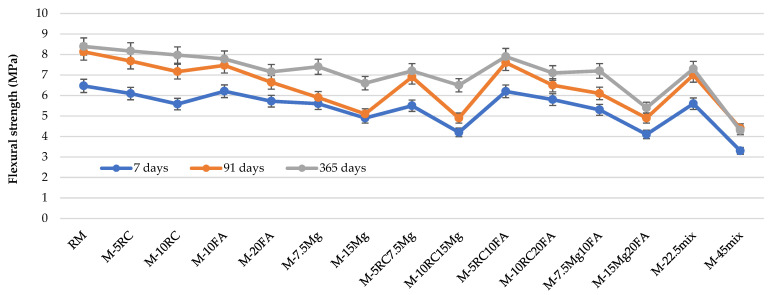
Flexural strength between 7 and 365 days of curing.

Figure 3 and Table 7 show the flexural strength at 7, 91, and 365 days. Comparing these results with those obtained in the compressive strength test, it can be concluded that in both tests, a similar behaviour of the different mortars can be observed.

At 91 days of curing, it was found that the binary mortars with 5% and 10% of RC showed a slight reduction in the value of the flexural capacity of 5.6% and 11.9%, respectively, compared to RM. With the use of 10% and 20% FA, a decrease of 8.1% and 18.3%, respectively, was obtained. Incorporating MgO in 7.5% and 15%, replacing OPC, a decrease in the mentioned capacity of 27.4% and 37.3%, respectively, was observed compared to the conventional mortar.

Regarding ternary mortars, it is concluded that in all mixes, the actual variation obtained in the laboratory, compared to RM, was lower than the theoretically expected variation. These findings prove the better behaviour of these materials in ternary mortars. These results can be justified by the fact that RC complements the reactivity of FA [41]. Additionally, when MgO is present in the mix, it can also react with the C-S-H gel, originated from FA or RC, to form magnesium silicate hydrate (M-S-H) gel, a compound that also has mechanical resistance [20].

For quaternary mortars, a reduction in flexural resistant capacity was also observed, with M-22.5mix having a 12.7% reduction, while in M-45mix the reduction reached 45.9%, when compared to RM. About the M-22.5mix mortar, it is possible to observe that the effect actually obtained in the experimental campaign is almost four times lower than the sum of the individual effects of each material in binary mortars, promoting the improvement in capacity when all materials are used together. This happens due the formation of multiple gels, including C-S-H, M-S-H, and M-A-H, which can contribute to the strength of the mortar [20,23].

The decrease in flexural values at 365 days in ternary mortars, relative to RM, was smaller than the decrease at 91 days (Figure 3). However, Table 7 shows that the differences between the variations of the results obtained in the laboratory and those theoretically expected were greater at 91 days. In other words, although the decrease is smaller at 365 days (compared to RM), it seems that the synergy between the materials works better at 91 days.

**Figure 4:**
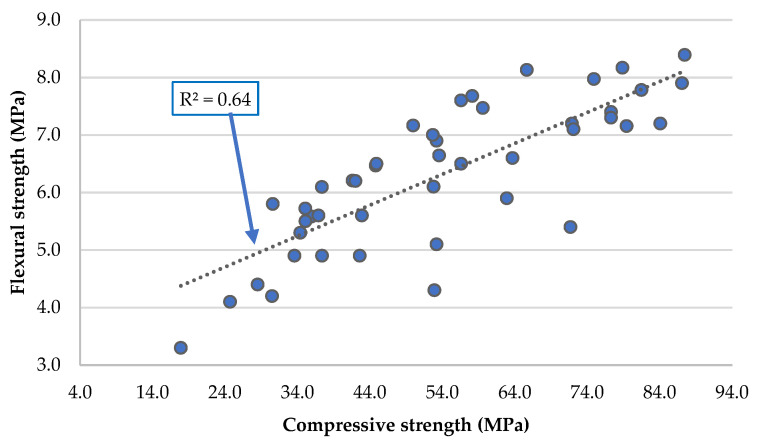
Relationship between flexural strength and the compressive strength (at the same age).

It is common to express the flexural strength results as a function of the compressive strength results. Observing Figure 4, there is a linear relationship between flexural strength and compressive strength, with R^2^ = 0.64. It can be concluded that the incorporation of RC, FA, and MgO, either individually or together, does not affect this usual relationship of the conventional mortars [52].

#### 3.2.4. Dynamic Modulus of Elasticity

**Figure 5:**
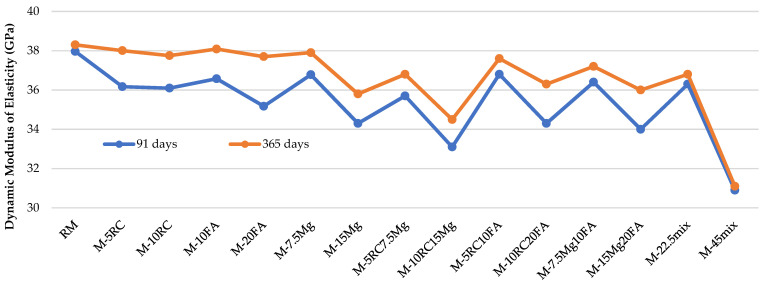
Dynamic modulus of elasticity at 91 and 365 days.

The results obtained for the dynamic modulus of the elasticity test can be seen in Table 8 and Figure 5. For the 91 day-old specimens, the E_c,dyn_ (dynamic modulus of elasticity) values vary between 30.9 GPa and 38.0 GPa. Highlighting the binary mortars, incorporating RC at 5% and 10%, there was a decrease of 4.7% and 4.9%, respectively, compared to the RM. As previously mentioned, these results are due to the dilution effect of RC [10]. Incorporating 10% and 20% of FA, there was a decrease of 3.7% and 7.4%, respectively, compared to RM. Regarding the latter material, the incorporation of 7.5% and 15% of MgO caused a decrease of 3.1% and 9.7% of the dynamic modulus of elasticity values, respectively. Although this decrease is not significant, it could be explained by the porosity increase due to the incorporation of MgO [19,22].

Note that in all the ternary mortars, the theoretical expected value was higher than the value obtained experimentally. These results show the good synergy between the materials when simultaneously incorporated into ternary mortars. This is because MgO can react with Ca(OH)_2_ to form magnesium hydroxide (Mg(OH)_2_) and calcium magnesium hydroxide (CaMg(OH)_4_) [20,21]. These products can also react with the C-S-H gel to form additional C-S-H gel, resulting in a better modulus of elasticity. Regarding the joint incorporation of all materials, the reduction in E_c,dyn_ varied between 4.4% and 18.6%, for M-22.5mix and M-45mix, respectively. This reduction is smaller than the sum of the effects in the individual mortars of each of the materials. This means that, with all the materials combined, there is also a positive synergistic effect between these different alternatives.

It is important to note that in all mixes, there were decreases in this property, relative to RM, that were lower than the amount of OPC substituted, attesting that the long life of the pozzolanic reactions balances the loss caused by the replacement of OPC.

Observing the values obtained at 365 days of curing, it can be seen that they are higher than those recorded at 91 days, with the minimum value of 31.1 GPa (M-45mix) and the maximum value of 38.3 GPa (RM). It was also concluded that from 91 to 365 days, there was an improvement in this capacity, comparing the sustainable mortars with RM. This lower variation relative to RM at 365 days is due to the new hydration reactions [20,21] that occur at later ages in these sustainable materials used (RC, FA, and MgO) [53,54].

**Figure 6:**
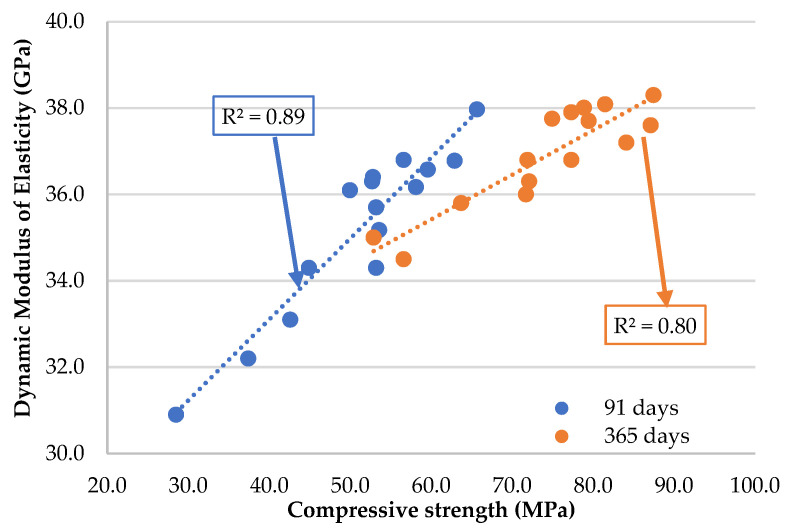
Dynamic modulus of elasticity at 91 and 365 days as a function of compressive strength (at the same age).

Finally, Figure 6 shows a linear relationship between the dynamic modulus of elasticity and the compressive strength in the mortars analysed. This strong linear relationship, usual in the conventional mortars, is proved by the R^2^ coefficients of 0.89 and 0.80, at 91 and 365 days, respectively [49].

#### 3.2.5. Ultrasonic Pulse Velocity

**Figure 7:**
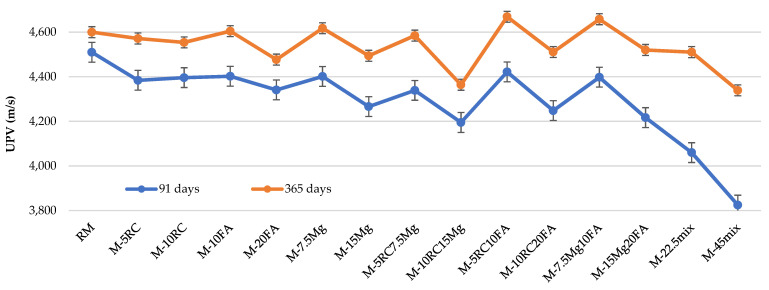
Ultrasonic pulse velocity at 91 and 365 days.

Table 9 and Figure 7 allow analysing the ultrasonic pulse velocity values at 91 and 365 days of curing of the different mortars produced. As expected, there is an increase in UPV with the age of the mixes. This is due to the filling of internal pores of the cementitious matrices with the formation of new hydration products of the binders [55].

Focusing on the 91-day results, values ranging between 3.8 km/s and 4.5 km/s were observed. At the level of binary mortars, the incorporation of RC in 5% and 10% causes a decrease of 2.8% and 2.5% compared to RM, respectively, justified by the dilution effect of RC. The use of FA (at 10% and 20%) causes a decrease in UPV between 2.4% and 3.7%, because the clinker dilution effect still prevailed over the FA’s pozzolanicity. The use of MgO in ratios of 7.5% and 15% causes a decrease of 1.9% and 6.1%, respectively, compared to conventional mortar, due the expansion effect of this material.

Again, relatively smaller decreases than theoretically expected were obtained in all ternary mortars. In fact, there were decreases between 2% (M-5RC10FA) and 7% (M-10RC15Mg). These decreases are notably lower than those recorded in the previous properties.

On the other hand, the combined incorporation of these materials caused a decrease of 10% and 15% in mortars M-22.5mix and M-45mix, respectively. In these mixes, unlike the ternary mixes, there was no good synergy between all the materials.

It is important to note that in all mixes, there were decreases in this property, relative to RM, that were lower than the amount of OPC substituted.

Naturally, there was an improvement in this property between 91 and 365 days of curing. A 2% increase in UPV values from 91 to 365 days was observed in RM. However, it should be noted that this improvement is more significant in the mortars produced with the alternative materials than in the reference mortar. In ternary mortars, there was an increase in UPV from 91 to 365 days between 4% and 7.2%. In quaternary mixes, the increase was higher, being 11.1% in M-22.5mix and 13.4% in M-45mix. This behaviour is due to the increased reactivity of FA in the presence of RC, causing denser microstructures compared to their binary counterparts that only had FA or RC. In addition, the expansive capacity of MgO, and the way it reacts when FA and/or RC are present, allows a formation of a denser microstructure at later ages.

These results presented fit with those obtained in the property of compressive strength and the dynamic modulus of elasticity.

It is argued by several authors [56,57] that UPV values higher than 4.5 km/s correspond to excellent quality mortars, and values between 3.6 km/s and 4.5 km/s are associated with good quality mortars. Table 6 allows concluding that, at 365 days, all the produced mortars, except for M-45mix and M-10RC15Mg, are classified as excellent quality mortars. Even these two exceptions present a UPV value of 4.3 km/s, close to the threshold to be considered an excellent quality mortar.

**Figure 8:**
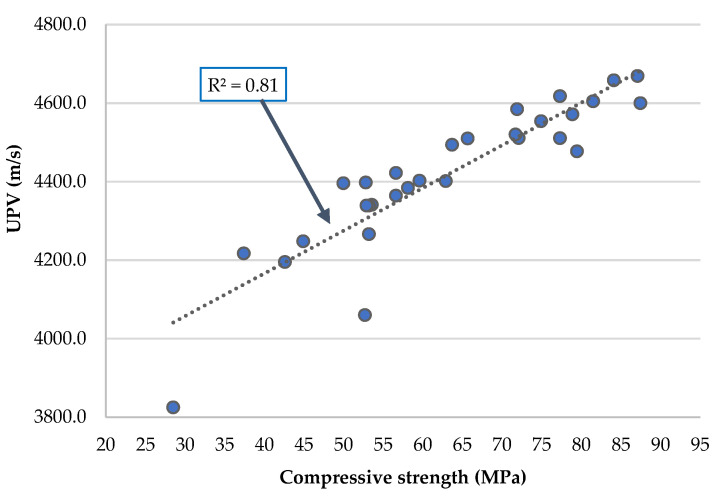
Ultrasonic pulse velocity at 91 and 365 days of curing as a function of the compressive strength, f_cm_, at the same age.

Figure 8 shows a linear increase in the compressive strength with UPV (R^2^ greater than 0.81).

#### 3.2.6. Eco-Efficient Material

Table 10 shows the estimated values of energy consumed for the production of 1 tonne of binder. Regarding OPC, the energy consumption values were obtained by analysing the available literature [58]. RC does not need calcination, but was crushed, so it was assumed that the energy consumed in the crushing process would be similar to the energy required to crush the CDW concrete debris [42]. The FA values were found in the available literature [59]. Finally, for MgO, a value of energy consumed obtained during investigations of other authors [60] was assumed.

**Figure 9:**
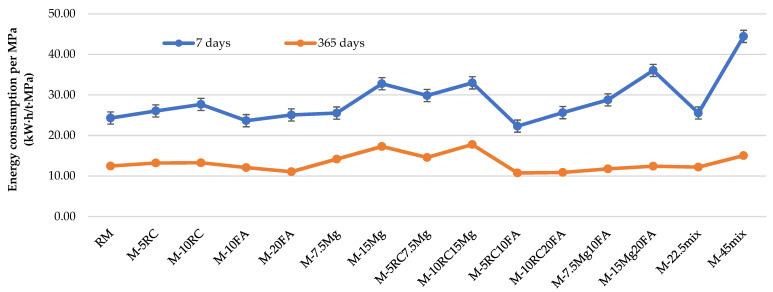
Energy performance at 7 days and 365 days of mortars.

The values of energy consumed per MPa are represented in Figure 9 and Table 11. At 7 days, the binary mortar with the highest energy consumption per MPa is M-15Mg. This is due to the higher energy consumption to manufacture one tonne of this material, combined with the lower compressive strength found, compared to RM. On the other hand, the mixes that present lower values of energy consumed are the binary mortars with FA, because the clinker dilution effect still prevails over the pozzolanic reactions [55]. The use of RC leads to an increase in the values obtained, compared to RM, due to the lower compressive strength at this age, and to the low reactivity of the supplementary cementitious materials [6].

Regarding the ternary mortars at 7 days, only in M-5RC10FA, there was a decrease (8.2%) in the energy consumed per MPa of strength, because the clinker dilution effect still prevails over the pozzolanic reactions [40]. In fact, the use of MgO in ternary mixes with FA or RC causes an increase in the values obtained between 18.4% (M-7.5Mg10FA) and 48.3% (M-15Mg20FA). This is largely due to the higher energy consumed during MgO fabrication compared to the other materials.

Among all the mixes produced, the quaternary mortar M-45mix presents the higher value, largely due to its lower compressive strength at 7 days, compared to the others.

At 365 days of age, the energy consumed per MPa is lower than that observed at 7 days, due to the gain in compressive strength of the mortars during this period. The lowest amount of energy consumed per MPa was observed in the binary mortars with FA.

In the ternary mortars, the decrease in consumed energy per MPa of strength can reach up to 13.7%, relative to the mortar with OPC, with simultaneous incorporation of 5% of RC and 10% of FA.

In the quaternary mortars, M-22.5mix and M-45mix, the energy consumed per MPa is 2.1% lower and 20.7% higher than in RM, respectively. The result of the M-22.5mix quaternary mortar highlights the improvement in capacity when all materials are used together. This means that replacing 22.5% of OPC with these materials decreased the energy consumed per MPa of force (at 365 days) by 2.1%.

In most results, the values obtained in the laboratory were better than those that could be expected, showing the good synergy between the sustainable materials when analysing this property.

Finally, it should be noted that the incorporation of RC [42], FA [57], and MgO [30,61], in partial replacement of OPC, leads to a decrease in CO_2_ emissions, as found by several authors [62].

## 4. Conclusions

In summary, after the analysis and discussion of the results obtained for all the mortars produced, several conclusions can be drawn.

In terms of the properties of concrete in the fresh state, it was found that the use of up to 10% of RC caused a slight reduction in workability (4.1%), compared to RM. The use of FA caused an increase in consistency between 7.7% and 17.7%, compared to RM. The incorporation of MgO caused a decrease in workability of a maximum of 8.9%. Simultaneous incorporations in ternary mortars caused variations between −13.7% and +11.1%. In quaternary mortars, a decrease between 2.2% and 2.6% was identified, relative to RM.

In all binary mortars produced, there was a slight decrease in their density relative to the reference mortar. Ternary and quaternary mortars also presented slight reductions, but lower than the sum of the effects of binary mortars.

Regarding the hardened state of mortars, a decrease in compressive strength was obtained in all mixes produced with sustainable materials compared to RM. At 365 days, M-5RC10FA obtained a decrease of only 0.4% (relative to RM). In all ternary mortars, experimentally obtained variations were better than those theoretically expected. This indicates a good synergistic relationship between the different materials. In terms of the quaternary mortars, the use of combined materials caused a reduction of 11.6% (M-22.5mix) and 39.5% (M-45mix) in the compressive strength at 365 days of curing.

Concerning the flexural strength at 365 days, there was also a reduction in the values obtained in this property for quaternary mixes, of 16.6% (M-22.5mix) and 48.8% (M-45mix), relative to RM. As with compressive strength, for the ternary mortars, there was also a good synergistic relationship between the different materials. There were decreases between 5.8% (M-5CR10FA) and 35.6% (M-15Mg20FA), compared to RM.

There was again a slight reduction in the dynamic modulus of elasticity in all alternative mortars compared to RM. However, except for M-45mix, at 365 days of curing, the maximum reduction was 9.9% (M-10RC15Mg) compared to RM. For example, in the M-22.5mix mortar, the reduction in this property was only 3.9%.

The incorporation of RC, FA, and MgO in ternary and quaternary mortars did not change the exponential correlation between ultrasonic pulse velocity and compressive strength, as established for other cementitious materials. It was concluded that in none of these mechanical properties was there any accumulation of the negative effects.

The energy consumed per MPa at 365 days of age showed better results in the ternary mortars with FA. The use of this material caused a decrease between 0.3% (M-15Mg20FA) and 13.7% (M-5RC10FA), compared to RM. The quaternary mortar M-22.5mix showed a reduction of 2.1%, while M-45mix led to an increase of 20.7%, relative to RM. The results allow for confirming that the simultaneous incorporation of RC, FA, and Mg, in ternary and quaternary mortars, can help achieve higher levels of cement replacements, especially when FA is used.

In summary, analysing the results of the mechanical properties and the energy performance, it is possible to conclude that the ideal ratio to use all these materials together, in partial replacement of cement, is 22.5%. The results of the homologous M-45mix originated much worse results than the M-22.5mix, not justifying its use. About the ternary mortars, the one that presented the best results was the one that incorporated RC and F, in the different ratios of incorporation.

To determine if recycled cement, fly ash, and magnesium oxide are suitable to use in concrete structures, further investigations into properties beyond just mechanical performance are required. These properties include durability, resistance to chemical attack, thermal performance, sound insulation, and fire resistance. By evaluating these properties, a better understanding of the overall suitability of these materials for different applications can be obtained.

By incorporating these sustainable materials into building projects, one can reduce the amount of waste generated, lower the carbon footprint associated with the production of new concrete, and reduce the amount of energy required for manufacturing. Consequently, this can contribute to mitigating the negative effects of climate change.

Furthermore, the use of sustainable materials in construction can help promote circularity within the industry. Instead of relying solely on extracting and consuming finite resources, this sector can move towards a more sustainable model that prioritizes the use of recycled and renewable materials.

## Figures and Tables

**Table 1:** Chemical composition of the materials (XRF test).

	Materials					
	Binders				Sands	
Oxides	OPC (%)	MgO (%)	RC (%)	FA (%)	Fine (%)	Coarse (%)
Mg	1.9	84.9	0.5	1.8	0.1	0.0
Al_2_O_3_	5.3	1.6	3.9	24.4	7.0	3.9
SiO_2_	19.3	6.5	47.5	57.5	87.2	92.8
CaO	67.1	3.1	41.2	7.1	0.2	0.2
Fe_2_O_3_	2.7	3.3	1.5	2.6	0.4	0.5
SO_3_	3.1	0.0	0.4	0.8	0.0	0.0
Other oxides	0.6	0.6	5.0	5.8	5.1	2.6

Note: OPC—ordinary Portland cement, RC—recycled cement, FA—fly ash, and MgO—magnesium oxide.

**Table 2:** Size and density of the binders.

Materials	Sieve Size (µm)			Percent Passing (%)	Specific Surface (m^2^/g)	Density (g/cm^3^)
	10	50	90	<63 µm		
OPC	2	14	46	97	2.8	3.1
FA	1	8	44	97	3.5	2.4
RC	2	21	147	68	1.1	2.5
MgO	9	129	310	29	4.9	3.1

**Table 3:** Composition of the RM.

Material	Content (kg/m^3^)
OPC	612
Other binders	0
Fine sand	641
Coarse sand	641
Total water	306

**Table 4:** Fresh-state properties of the mortars.

Mortar Mix	Consistency (mm)	Air Content (vol %)	Density (kg/m^3^)
RM	271	1.6	2249
M-5RC	268	1.9	2221
M-10RC	260	2.2	2213
M-10FA	292	1.0	2230
M-20FA	319	0.8	2203
M-7.5Mg	258	2.1	2228
M-15Mg	247	2.8	2193
M-5RC7.5Mg	265	2.2	2221
M-10RC15Mg	234	2.8	2202
M-5RC10FA	284	1.0	2240
M-10RC20FA	301	0.7	2217
M-7.5Mg10FA	280	1.1	2224
M-15Mg20FA	243	1.2	2203
M-22.5mix	265	1.2	2230
M-45mix	264	1.0	2212

**Table 5:** Compressive strength at 91 and 365 days of curing.

Mortar Mix	Total OPC Replacement (%)	91 Days			365 Days		
		Comp. (MPa)	△ Real (%)	△ Theoretical (%)	Comp. (MPa)	△ Real (%)	△ Theoretical (%)
RM	-	65.7	-	-	87.5	-	-
M-5RC	5.0	58.1	−11.5	-	78.9	−9.8	-
M-10RC	10.0	50.0	−23.9	-	74.9	−14.3	-
M-10FA	10.0	59.6	−9.2	-	81.5	−6.8	-
M-20FA	20.0	53.6	−18.4	-	79.4	−9.2	-
M-7.5Mg	7.5	62.9	−4.2	-	77.3	−11.6	-
M-15Mg	15.0	53.2	−19.0	-	63.7	−27.2	-
M-5RC7.5Mg	12.5	53.2	−19.0	−15.7	71.9	−17.8	−21.4
M-10RC15Mg	25.0	42.6	−35.1	−42.9	56.6	−35.3	−41.5
M-5RC10FA	15.0	56.6	−13.8	−20.7	87.1	−0.4	−16.6
M-10RC20FA	30.0	44.9	−31.6	−42.3	72.1	−17.6	−23.5
M-7.5Mg10FA	17.5	52.8	−19.6	−13.4	84.1	−3.8	−18.4
M-15Mg20FA	35.0	37.4	−43.0	−37.4	71.7	−18.0	−36.4
M-22.5mix	22.5	52.7	−19.7	−24.9	77.3	−11.6	−28.2
M-45mix	45.5	28.5	−56.6	−61.3	52.9	−39.5	−50.7

Note: △ expected theoretical of each ternary and quaternary mix corresponds to the sum of △ real of the binary mixes with the correspondent materials.

**Table 6:** Strength activity index between 7 and 365 days of curing.

Mortar Mix	7 Days	91 Day	365 Days
	SAI (%)	SAI (%)	SAI (%)
RM	-	-	-
M-5RC	83.4	88.5	90.2
M-10RC	80.2	76.1	85.7
M-10FA	92.9	90.8	93.2
M-20FA	78.3	81.6	90.8
M-7.5Mg	95.7	95.8	88.4
M-15Mg	75.0	81.0	72.8
M-5RC7.5Mg	78.3	81.0	82.2
M-10RC15Mg	68.1	64.9	64.7
M-5RC10FA	93.7	86.2	99.6
M-10RC20FA	68.3	68.4	82.4
M-7.5Mg10FA	76.8	80.4	96.2
M-15Mg20FA	55.1	57.0	82.0
M-22.5mix	82.3	80.3	88.4
M-45mix	39.9	43.4	60.5

**Table 7:** Flexural strength between 91 and 365 days of curing.

Mortar Mix	Total OPC Replacement (%)	91 Days			365 Days		
		Flexural. (MPa)	△ Real (%)	△ Theoretical (%)	Flexural (MPa)	△ Real (%)	△ Theoretical (%)
RM	-	8.1	-	-	8.4	-	-
M-5RC	5.0	7.7	−5.6	-	8.1	−2.6	-
M-10RC	10.0	7.2	−11.9	-	8.0	−5.0	-
M-10FA	10.0	7.5	−8.1	-	7.8	−7.2	-
M-20FA	20.0	6.6	−18.3	-	7.2	−14.7	-
M-7.5Mg	7.5	5.9	−27.4	-	7.4	−11.8	-
M-15Mg	15.0	5.1	−37.3	-	6.6	−21.3	-
M-5RC7.5Mg	12.5	6.9	−15.1	−33.0	7.2	−14.2	−14.4
M-10RC15Mg	25.0	4.9	−39.7	−49.2	6.5	−22.5	−26.3
M-5RC10FA	15.0	7.6	−6.5	−13.7	7.9	−5.8	−9.8
M-10RC20FA	30.0	6.5	−20.1	−30.2	7.1	−15.4	−19.7
M-7.5Mg10FA	17.5	6.1	−25.0	−35.5	7.2	−14.2	−19.0
M-15Mg20FA	35.0	4.9	−39.7	−55.6	5.4	−35.6	−36.0
M-22.5mix	22.5	7.1	−12.7	−41.1	7.0	−16.6	−21.6
M-45mix	45.5	4.4	−45.9	−67.5	4.3	−48.8	−41.0

Note: △ expected theoretical of each ternary and quaternary mix correspond to the sum of △ real of the binary mixes with the correspondent materials.

**Table 8:** Dynamic modulus of elasticity at 91 and 365 days.

Mortar Mix	Total OPC Replacement (%)	91 Days			365 Days		
		Modulus of Elasticity (MPa)	△ Real (%)	△ Theoretical (%)	Modulus of Elasticity (MPa)	△ Real (%)	△ Theoretical (%)
RM	-	38.0	-	-	38.3	-	-
M-5RC	5.0	36.2	−4.7	-	38.0	−0.8	-
M-10RC	10.0	36.1	−4.9	-	37.8	−1.4	-
M-10FA	10.0	36.6	−3.7	-	38.1	−0.6	-
M-20FA	20.0	35.2	−7.4	-	37.7	−1.6	-
M-7.5Mg	7.5	36.8	−3.1	-	37.9	−1.0	-
M-15Mg	15.0	34.3	−9.7	-	35.8	−6.5	-
M-5RC7.5Mg	12.5	35.7	−6.0	−7.8	36.8	−3.9	−1.8
M-10RC15Mg	25.0	33.1	−12.8	−14.6	34.5	−9.9	−7.9
M-5RC10FA	15.0	36.8	−3.1	−8.4	37.6	−1.8	−1.4
M-10RC20FA	30.0	34.3	−9.7	−12.3	36.3	−5.2	−3.0
M-7.5Mg10FA	17.5	36.4	−4.1	−6.8	37.2	−2.9	−1.6
M-15Mg20FA	35.0	34.0	−10.4	−17.1	36.0	−6.0	−8.1
M-22.5mix	22.5	36.3	−4.4	−11.5	36.8	−3.9	−2.4
M-45mix	45.5	30.9	−18.6	−22.0	31.1	−18.6	−9.5

Note: △ expected theoretical of each ternary and quaternary mix corresponds to the sum of △ real of the binary mixes with the correspondent materials.

**Table 9:** Ultrasonic pulse velocity between 91 and 365 days.

Mortar Mix	Total OPC Replacement (%)	91 Days			365 Days		
		UPV (m/s)	△ Real (%)	△ Theoretical (%)	UPV (m/s)	△ Real (%)	△ Theoretical (%)
RM	-	4510	-	-	4600	-	-
M-5RC	5.0	4384	−2.8	-	4572	−0.6	-
M-10RC	10.0	4396	−2.5	-	4554	−1.0	-
M-10FA	10.0	4402	−2.4	-	4605	+0.1	-
M-20FA	20.0	4341	−3.7	-	4477	−2.7	-
M-7.5Mg	7.5	4401	−2.4	-	4618	+0.4	-
M-15Mg	15.0	4266	−5.4	-	4494	−2.3	-
M-5RC7.5Mg	12.5	4339	−3.8	−5.2	4585	−0.3	−0.2
M-10RC15Mg	25.0	4195	−7.0	−7.9	4364	−5.1	−3.3
M-5RC10FA	15.0	4422	−2.0	−5.2	4669	+1.5	−0.5
M-10RC20FA	30.0	4248	−5.8	−6.2	4511	−1.9	−3.7
M-7.5Mg10FA	17.5	4398	−2.5	−4.8	4658	+1.3	+0.5
M-15Mg20FA	35.0	4217	−6.5	−9.1	4520	−1.7	−5.0
M-22.5mix	22.5	4060	−10.0	−7.6	4511	−1.9	−0.1
M-45mix	45.5	3825	−15.2	−11.6	4339	−5.7	−6.0

Note: △ expected theoretical of each ternary and quaternary mix corresponds to the sum of △ real of the binary mixes with the correspondent materials.

**Table 10:** Values used to calculate the energy consumed.

Material	Energy Consumed
OPC	1089 kW·h/t
FA	38 kW·h/t
RC	140 kW·h/t
MgO	1165 kW·h/t

**Table 11:** Energy performance at 7 days and 365 days of mortars.

Mortar Mix	Total OPC Replacement (%)	7 Days			365 Days		
		E (kW·h/t·MPa)	△ Real (%)	△ Theoretical (%)	E (kW·h/t·MPa)	△ Real (%)	△ Theoretical (%)
RM	-	24.30	-	-	12.45	-	-
M-5RC	5.0	26.04	+7.2	-	13.21	+6.1	-
M-10RC	10.0	27.65	+13.8	-	13.27	+6.5	-
M-10FA	10.0	23.64	−2.7	-	12.07	−3.0	-
M-20FA	20.0	25.04	+3.1	-	11.06	−11.2	-
M-7.5Mg	7.5	25.52	+5.0	-	14.16	+13.7	-
M-15Mg	15.0	32.75	+34.8	-	17.27	+38.7	-
M-5RC7.5Mg	12.5	29.84	+22.8	+12.2	14.57	+17.0	+19.8
M-10RC15Mg	25.0	32.97	+34.7	+48.6	17.77	+42.7	+45.2
M-5RC10FA	15.0	22.30	−8.2	+4.5	10.75	−13.7	+3.1
M-10RC20FA	30.0	25.62	+5.4	+16.9	10.87	−12.7	−4.7
M-7.5Mg10FA	17.5	28.77	+18.4	+8.1	11.77	−5.5	+10.7
M-15Mg20FA	35.0	36.04	+48.3	+37.9	12.42	−0.3	+27.5
M-22.5mix	22.5	25.53	+5.1	+9.5	12.19	−2.1	+16.8
M-45mix	45.5	44.43	+82.8	+51.7	15.03	+20.7	+34.0

Note: △ expected theoretical of each ternary and quaternary mix corresponds to the sum of △ real of the binary mixes with the correspondent materials.

## Data Availability

Not applicable.

## References

[1] Benhelal E., Zahedi G., Shamsaei E., Bahadori A. (2013). Global strategies and potentials to curb CO_2_ emissions in cement industry. J. Clean. Prod..

[2] De Schepper M., Van den Heede P., Van Driessche I., De Belie N. (2014). Life cycle assessment of completely recyclable concrete. Materials.

[3] Sun L., Wang C., Zhang C., Yang Z., Li C., Qiao P. (2023). Experimental investigation on the bond performance of sea sand coral concrete with FRP bar reinforcement for marine environments. Adv. Struct. Eng..

[4] Wang B., Yan L., Fu Q., Kasal B. (2021). A comprehensive review on recycled aggregate and recycled aggregate concrete. Resour. Conserv. Recycl..

[5] Bai B., Bai F., Nie Q., Jia X. (2023). A high-strength red mud–fly ash geopolymer and the implications of curing temperature. Powder Technol..

[6] Kim Y.-J. (2017). Quality properties of self-consolidating concrete mixed with waste concrete powder. Constr. Build. Mater..

[7] Xiao J., Ma Z., Sui T., Akbarnezhad A., Duan Z. (2018). Mechanical properties of concrete mixed with recycled powder produced from construction and demolition waste. J. Clean. Prod..

[8] Oliveira T.C.F., Dezen B.G.S., Possan E. (2020). Use of concrete fine fraction waste as a replacement of Portland cement. J. Clean. Prod..

[9] Moreno-Juez J., Vegas I.J., Frías Rojas M., Vigil de la Villa R., Guede-Vázquez E. (2021). Laboratory-scale study and semi-industrial validation of viability of inorganic CDW fine fractions as SCMs in blended cements. Constr. Build. Mater..

[10] Kim Y.J., Choi Y.W. (2012). Utilization of waste concrete powder as a substitution material for cement. Constr. Build. Mater..

[11] Kwon E., Ahn J., Cho B., Park D. (2015). A study on development of recycled cement made from waste cementitious powder. Constr. Build. Mater..

[12] Kalinowska-Wichrowska K., Pawluczuk E., Bołtryk M., Jimenez J.R., Fernandez-Rodriguez J.M., Suescum Morales D. (2022). The performance of concrete made with secondary products-recycled coarse aggregates, recycled cement mortar, and fly ash-slag mix. Materials.

[13] Garcia-Lodeiro I., Carcelen-Taboada V., Fernández-Jiménez A., Palomo A. (2016). Manufacture of hybrid cements with fly ash and bottom ash from a municipal solid waste incinerator. Constr. Build. Mater..

[14] Ameri F., Zareei S.A., Behforouz B. (2020). Zero-cement vs. cementitious mortars: An experimental comparative study on engineering and environmental properties. J. Build. Eng..

[15] Fu X., Wang Z., Tao W., Yang C., Hou W., Dong Y., Wu X. (2002). Studies on blended cement with a large amount of fly ash. Cem. Concr. Res..

[16] Malek R.I., Khalil Z.H., Imbaby S.S., Roy D.M. (2005). The contribution of class-F fly ash to the strength of cementitious mixtures. Cem. Concr. Res..

[17] Massazza F., Hewlett P.C. (1998). 10-Pozzolana and pozzolanic cements. Lea’s Chemistry of Cement and Concrete.

[18] Mo L., Liu M., Al-Tabbaa A., Deng M. (2015). Deformation and mechanical properties of the expansive cements produced by inter-grinding cement clinker and MgOs with various reactivities. Constr. Build. Mater..

[19] Gonçalves T., Silva R.V., de Brito J., Fernández J.M., Esquinas A.R. (2020). Mechanical and durability performance of mortars with fine recycled concrete aggregates and reactive magnesium oxide as partial cement replacement. Cem. Concr. Compos..

[20] Vandeperre L.J., Liska M., Al-Tabbaa A. (2008). Microstructures of reactive magnesia cement blends. Cem. Concr. Compos..

[21] Brew D.R.M., Glasser F.P. (2005). Synthesis and characterisation of magnesium silicate hydrate gels. Cem. Concr. Res..

[22] Ye G., Troczynski T. (2006). Hydration of hydratable alumina in the presence of various forms of MgO. Ceram. Int..

[23] Choi S.-w., Jang B.-s., Kim J.-h., Lee K.-m. (2014). Durability characteristics of fly ash concrete containing lightly-burnt MgO. Constr. Build. Mater..

[24] Sánchez Berriel S., Favier A., Rosa Domínguez E., Sánchez Machado I.R., Heierli U., Scrivener K., Martirena Hernández F., Habert G. (2016). Assessing the environmental and economic potential of limestone calcined clay cement in Cuba. J. Clean. Prod..

[25] Abdalqader A., Al-Tabbaa A. Mechanical and Microstructural Characterisation of Multicomponent Blended Cements Incorporating Reactive Magnesia. Proceedings of the 1st Concrete Innovative Conference (CIC).

[26] Chen X., Li Y., Bai H., Ma L. (2021). Utilization of recycled concrete powder in cement composite: Strength, microstructure and hydration characteristics. J. Renew. Mater..

[27] Cuenca-Moyano G.M., Martín-Pascual J., Martín-Morales M., Valverde-Palacios I., Zamorano M. (2020). Effects of water to cement ratio, recycled fine aggregate and air entraining/plasticizer admixture on masonry mortar properties. Constr. Build. Mater..

[28] Jiang D., Li X., Lv Y., Zhou M., He C., Jiang W., Liu Z., Li C. (2020). Utilization of limestone powder and fly ash in blended cement: Rheology, strength and hydration characteristics. Constr. Build. Mater..

[29] Mavroulidou M., Morrison T., Unsworth C., Gunn M. (2015). Properties of concrete made of multicomponent mixes of low-energy demanding binders. Constr. Build. Mater..

[30] Unluer C., Al-Tabbaa A. (2013). Impact of hydrated magnesium carbonate additives on the carbonation of reactive MgO cements. Cem. Concr. Res..

[31] Cantero B., Bravo M., de Brito J., del Bosque I.F., Medina C. (2022). The influence of fly ash on the mechanical performance of cementitious materials produced with recycled cement. Appl. Sci..

[32] (2014). Tests for Mechanical and Physical Properties of Aggregates. Part 6: Determination of Particle Density and Water Absorption.

[33] Faury J., Dunod P. (1958). Le Béton.

[34] (2013). Part 1: Specification, Production and Conformity.

[35] (1999). Methods of Test for Mortar for Masonr. Part 3: Determination of Consistence of Fresh Mortar.

[36] (1999). Methods of Test for Mortar for Masonr. Part 6: Determination of Bulk Density of Fresh Mortar.

[37] (2019). Testing Fresh Concrete. Part 7: Air Conten. Pressure Methods.

[38] (2020). Methods of Test for Mortar for Masonr. Part 11: Determination of Flexural and Compressive Strength of Hardened Mortar.

[39] (2016). Standard Test Method for Dynamic Young’s Modulus, and Poisson’s Ratio by Impulse Excitation of Vibration.

[40] (2004). Testing Concrete in Structures. Part 4: Determination of Ultrasonic Pulse Velocity.

[41] Hamidi M., Kacimi L., Cyr M., Clastres P. (2013). Evaluation and improvement of pozzolanic activity of andesite for its use in eco-efficient cement. Constr. Build. Mater..

[42] Cantero B., Bravo M., de Brito J., Sáez del Bosque I.F., Medina C. (2020). Mechanical behaviour of structural concrete with ground recycled concrete cement and mixed recycled aggregate. J. Clean. Prod..

[43] Duan Z., Hou S., Xiao J., Singh A. (2020). Rheological properties of mortar containing recycled powders from construction and demolition wastes. Constr. Build. Mater..

[44] Luo X., Gao J., Li S., Xu Z., Chen G. (2022). Experimental study on the early-age properties of cement pastes with recycled brick powder. Constr. Build. Mater..

[45] Jin F., Al-Tabbaa A. (2014). Strength and hydration products of reactive MgO–silica pastes. Cem. Concr. Compos..

[46] Demirboǧa R. (2003). Influence of mineral admixtures on thermal conductivity and compressive strength of mortar. Energy Build..

[47] Dhandapani Y., Santhanam M., Kaladharan G., Ramanathan S. (2021). Towards ternary binders involving limestone additions-A review. Cem. Concr. Res..

[48] Xu B., Lothenbach B., Ma H. (2018). Properties of fly ash blended magnesium potassium phosphate mortars: Effect of the ratio between fly ash and magnesia. Cem. Concr. Compos..

[49] Wang X.-Y., Lee H.-S. (2010). Modeling the hydration of concrete incorporating fly ash or slag. Cem. Concr. Res..

[50] (2014). Standard Specification for Coal Fly Ash and Raw or Calcined Natural Pozzolan for Use in Concrete.

[51] Horsakulthai V. (2021). Effect of recycled concrete powder on strength, electrical resistivity, and water absorption of self-compacting mortars. Case Stud. Constr. Mater..

[52] Dehghan S.M., Najafgholipour M.A., Baneshi V., Rowshanzamir M. (2019). Experimental study on effect of water–cement ratio and sand grading on workability and mechanical properties of masonry mortars in Iran. Iran. J. Sci. Technol. Trans. Civ. Eng..

[53] Cantero B., Sáez del Bosque I.F., Matías A., Sánchez de Rojas M.I., Medina C. (2020). Water transport mechanisms in concretes bearing mixed recycled aggregates. Cem. Concr. Compos..

[54] Paris J.M., Roessler J.G., Ferraro C.C., DeFord H.D., Townsend T.G. (2016). A review of waste products utilized as supplements to Portland cement in concrete. J. Clean. Prod..

[55] Bogas J., Gomes M., Gomes A. (2013). Compressive strength evaluation of structural lightweight concrete by non-destructive ultrasonic pulse velocity method. Ultrasonics.

[56] Alnahhal M.F., Alengaram U.J., Jumaat M.Z., Alqedra M.A., Mo K.H., Sumesh M. (2017). Evaluation of industrial by-products as sustainable pozzolanic materials in recycled aggregate concrete. Sustainability.

[57] Saint-Pierre F., Philibert A., Giroux B., Rivard P. (2016). Concrete quality designation based on ultrasonic pulse velocity. Constr. Build. Mater..

[58] Arribas I., Vegas I., García V., Vigil de la Villa R., Martínez-Ramírez S., Frías M. (2018). The deterioration and environmental impact of binary cements containing thermally activated coal mining waste due to calcium leaching. J. Clean. Prod..

[59] Hossain M.U., Poon C.S., Lo I.M.C., Cheng J.C.P. (2016). Comparative environmental evaluation of aggregate production from recycled waste materials and virgin sources by LCA. Resour. Conserv. Recycl..

[60] Ruan S., Unluer C. (2016). Comparative life cycle assessment of reactive MgO and Portland cement production. J. Clean. Prod..

[61] Liska M., Al-Tabbaa A. (2009). Ultra-green construction: Reactive magnesia masonry products. Waste Resour. Manag..

[62] Walling S.A., Provis J.L. (2016). Magnesia-based cements: A journey of 150 years, and cements for the future?. Chem. Rev..

